# Xenobiotics, Oxidative Stress, and Antioxidants

**DOI:** 10.1155/2018/9758951

**Published:** 2018-05-22

**Authors:** Fatma M. El-Demerdash, Ehab M. Tousson, Jacek Kurzepa, Samy L. Habib

**Affiliations:** ^1^Environmental Studies Department, Institute of Graduate Studies and Research, University of Alexandria, 163 Horrya Av., P.O. Box 832 El Shatby, Alexandria, Egypt; ^2^Zoology Department, Faculty of Science, Tanta University, Tanta, Egypt; ^3^Department of Medical Chemistry, Medical University of Lublin, Chodzki 4a, 20-093 Lublin, Poland; ^4^University of Texas Health, San Antonio, TX, USA

Human and animals are exposed to large number of biological and environmental hazards. Free radicals and oxidant species may act as deleterious and toxic products, involved in cellular and organ dysfunction. Overproduction of these species may result in DNA, lipid, and protein damage. Nevertheless, low or moderate concentrations of reactive oxygen species (ROS) or reactive nitrogen species (RNS) are also involved in physiological responses as part of signaling processes and defense mechanisms. Oxidative stress is arguably the most common mechanism in the toxicology of environmental agents. Cells are equipped with multiple complementary energy-dependent systems for maintaining redox homeostasis in the face of environmental oxidative stress. The cell has several means available to tackle free radical generation including antioxidants and antioxidant enzymes. There is a growing interest of natural products in human diet. Increased consumption of plant-based, antioxidant-rich foods including, fruits, vegetables, whole grains, and nuts is associated with the reduced risk of several chronic diseases. In addition, a great number of spices and aromatic herbs contain chemical compounds exhibiting antioxidant properties since they have a variety of active phytochemicals including vitamins, carotenoids, terpenoids, alkaloids, flavonoids, lignans, simple phenols, and phenolic acids. In this special issue on Xenobiotics, Oxidative Stress, and Antioxidants, five papers are included.

In this issue, K. Michalak et al. addressed that the main directions of possible effective treatment of fluoroquinolone-associated disability (FQAD) to reduce oxidative stress, restoring reduced mitochondrion potential, supplementation of uni- and bivalent cations that are chelated by FQs. FQAD may also ineffectively transported to the cell, stimulating the mitochondrial proliferation, removing fluoroquinolones permanently accumulated in the cells, and regulating the disturbed gene expression as well as enzyme activity. G. Murtaza et al. reported that neurodegeneration and cancer diseases are associated with aging, which is affected by many genetic and environmental factors. Healthy aging conceives human longevity, possibly due to carrying the defensive genes. FOXO (forkhead box O) genes are involved in the multiple cellular pathways, which regulate growth, stress resistance, metabolism, cellular differentiation, and apoptosis in mammals. D. Yuan et al. evaluated the antioxidant and anti-inflammatory effects of *Eucommia ulmoides flavones* (EUF) using diquat-challenged piglet models. They showed that EUF supplementation improved the growth performance of diquat-treated piglets from day 14 to 21. Diquat also induced oxidative stress and inflammatory responses and impaired intestinal morphology. EUF alleviated these negative effects induced by diquat to decease serum concentrations of proinflammatory cytokines and increase antioxidant indexes as well as anti-inflammatory cytokines on day 14. They indicated that EUF attenuated the inflammation and oxidative stress of piglets caused by diquat injection. T. Zhang et al. demonstrated that avenanthramides, a group of diphenolic acids found only in oats are bioavailable to human, have anti-inflammatory and antioxidant effects as a useful natural phytochemical in sports science and chronic disease prevention. L. Lin et al. showed that *N*-hydroxycinnamoylphenalkylamides (36H) is an effective skin-whitening agent that has the potential for cosmetic applications due to the biofunctions of antioxidation and melanin suppression.

*Fatma M. El-Demerdash* *Ehab M. Tousson* *Jacek Kurzepa* *Samy L. Habib*

